# Low Diagnostic Accuracy of Body Mass Index-Based and Waist Circumference-Based References of Childhood Overweight and Obesity in Identifying Overfat among Chinese Children and Adolescents

**DOI:** 10.1155/2018/4570706

**Published:** 2018-12-12

**Authors:** Yiyang Chen, Yu Zhang, Lin Wang

**Affiliations:** Shanghai University of Sport, Shanghai, China

## Abstract

This study aimed to investigate the diagnostic accuracy of body mass index- (BMI-) based and waist circumference- (WC-) based references for childhood overweight and obesity in screening overfat individuals among 2134 Chinese children and adolescents. In this study, overfat status was defined as over 25% body fat for boys and over 30% for girls. Childhood obesity or overweight was defined by four BMI-based references and two WC-based references. All BMI-based references for obesity showed low sensitivity (SE) (0.128–0.473) but high specificity (SP) (0.971–0.998) in detecting overfat individuals in the current population. SE values increased from 0.493 to 0.881 when BMI- and WC-based references for overweight were used to detect overfat individuals. All references for overweight showed high SP rates (0.816–0.966). To improve diagnostic accuracy for childhood obesity, further studies may define a cut-off value for childhood obesity specific for a local population and ethnicity by using health-related overfat data.

## 1. Introduction

Overweight and obesity are generally defined as abnormal or excessive fat accumulation that presents health risks [1]. The prevalence of overweight and obesity is greatly increasing from 6 years to 12 years in childhood, and most overweight or obese children present with cardiac abnormalities [2, 3]. In large-scale population surveys, various references for childhood overweight and obesity have been developed using anthropometry measurements. Body mass index (BMI) is an important measurement for defining obesity [4, 5]. BMI references from the World Health Organization (WHO), International Obesity Task Force (IOTF), and US Centers for Disease Control and Prevention (CDC) are three common BMI-based references for childhood overweight and obesity [6–8]. In China, a BMI-based reference for Chinese childhood overweight and obesity was developed by the Working Group on Obesity in China (WGOC) [9].

A pilot study suggests waist-to-height ratio (WHtR), a marker of central adiposity and BMI could enhance assessments of the effectiveness of childhood obesity in a two-year broad multicomponent educational program [10, 11]. Previous studies recommended that waist circumference (WC) and BMI produce similar results in identifying overfat, cardiometabolic, and multiple metabolic risk factors in children and elderly population [11–13]. Therefore, WC and waist-to-height ratio (WHtR) have attracted considerable attention as indicators of overfat status and health risks among children and adults [5, 14, 15]. WC-based references for childhood overweight and obesity have been developed. Studies have recommended several WC-based references for childhood overweight for Chinese children and adolescents [12, 16, 17].

BMI-based references for childhood obesity present poor diagnostic performance in identifying overfat individuals among Chinese children and adolescents [18]. On the other hand, for WC-based references for childhood overweight and obesity, the diagnostic performance in identifying overfat individuals in Chinese children remains unclear.

The current study aimed to investigate the diagnostic accuracy of BMI- and WC-based references for childhood overweight and obesity in screening overfat individuals and to determine the relationship between anthropometric measurements and body fat percentage (%BF) in Chinese children and adolescents.

## 2. Materials and Methods

### 2.1. Participants

Participants were healthy children, who completed a screening medical history and physical examination. Participants were excluded from analysis in children treated with lipid-, glucose-, and blood pressure-lowering medications. Subjects with morbid obesity who had symptoms with metabolic disturbances, cardiovascular abnormalities and diabetes were excluded from the participation. Convenience samples were recruited in local primary and secondary schools. After screening and excluding 57 participants with missing measurement data, the data on 1135 boys and 999 girls were analyzed. The current study was approved by the Joint Chinese University of Hong Kong–New Territories East Cluster Clinical Research Ethics Committee. Written informed consent was obtained from all participants and their parents (for participants under 18 years old).

### 2.2. Anthropometric Measurement

Anthropometric measurements, namely, body weight, body height, and waist circumference, were measured by two trained researchers. Body weight was measured using a scale (Tanita TBF-543, Tokyo, Japan) to the nearest 0.1 kg, and body height was measured using a portable stadiometer (Seca 213 portable stadiometer, Hamburg, Germany) to the nearest 0.5 cm with bare feet and minimal clothing. In our study, as recommended by World Health Organization and International Diabetes Federation, we used the midpoint between the lowest rib and superior border of the iliac crest, the most common used WC protocols (35%) as measurement site [19–21].WC was measured by using an inelastic measuring tape and recorded to the nearest 0.1 cm.

### 2.3. Body Fat Measurement

A foot-foot bioelectric impedance (BIA) analyzer (Tanita TBF-543, Tanita, Tokyo, Japan) was used to measure %BF. The device was used in our previous study [18]. The portable BIA scale performs well in measuring %BF in Chinese children [22]. BIA measurements were performed on an empty bladder at least 2 h after a meal. Gender, age, and body height were manually entered. The participants were requested to wear light sportswear and stand barefoot on four metal electrodes. Body weight was measured, and %BF was calculated using the embedded equations in the device.

### 2.4. Definition of Overfat

In the current study, overfat was defined as over 30% body fat for girls and over 25% body fat for boys [23]. The cut-offs are strongly associated with cardiovascular risk factors in children and adolescents and are therefore widely used to define overfat status [24–26].

### 2.5. Classification of Participants

The participants were classified according to four BMI-based references for childhood overweight and obesity, one WC reference, and one WHtR reference for childhood overweight. In the current study, the four BMI-based references used included IOTF BMI, CDC BMI, WHO BMI, and WGOC BMI for childhood overweight and obesity.

Ng et al. proposed optimal age- and gender-specific WC cut-offs to define childhood overweight and predict clustering of cardiovascular risk factors in Chinese children [17]. In their study, WC values higher than 76.8^th^ percentile for girls and 76.1^th^ percentile for boys predicted high cardiovascular risk in children. The present study also used the same cut-offs to identify children and adolescents with high WC. A study suggested a WHtR of over 0.46 as cut-off for overweight in Hong Kong Chinese children and adolescents [16]. This cut-off was also used to define childhood overweight in the current study.

### 2.6. Data Reduction and Statistical Analysis

Statistical analyses were performed using SPSS 17.0 software (SPSS Inc., Chicago, IL, USA). Mean and standard deviation (SD) summarized the continuous variables. Age-z score, BW-z score, BH-z score, WC-z score, WHtR-z score, BMI-z score, and %BF-z score were represented as median(range) [27, 28]. The differences in z scores among two genders were compared using Kruskal-Wallis one-way ANOVA.

Independent t-test was used to determine differences in between-gender comparisons in %BF and anthropometric measurements. The relationships between %BF and anthropometric measurements were evaluated using partial correlation coefficients adjusted by age. Chi-square tests were used to assess differences in prevalence of childhood overweight and obesity between boys and girls.

Diagnostic accuracy of BMI- and WC-based references in identifying overfat was evaluated by comparing their respective diagnostic accuracy indices. These indices included sensitivity (SE), specificity (SP), likelihood ratio for positive test results (LR+), positive predictive values (PPV), and Youden's index (YI).

## 3. Results

### 3.1. Characteristics of Participants

As shown in Table 1, the study population consisted of 2134 children and adolescents aged 9–19 years with 46.8% girls (age: 13.9 ± 2.7 years) and 53.2% boys (age: 13.7 ± 2.9 years). No significant differences were observed in the age-z-score, BW-z score, BH-z score, WC-z score, WHtR-z score, BMI-z score, and %BF-z score between boys and girls. Median age-z-score was significantly higher in 5th percentile and 95th percentile girls. Median BH-z-score was significantly higher in 95th percentile girls than boys. The BMI-z score and %BF-z score were significantly lower than those of 5th percentile boys (Table 1).

### 3.2. Age-Adjusted Correlations among the Different Indices of Obesity

BMI, WC, and WHtR correlated well with each other in boys (*r *= 0.877–0.977,* p *< 0.001) and girls (*r *= 0.745–0.932,* p *< 0.001) after adjusting for participants' age. The age-adjusted correlation coefficients between %BF and anthropometric measurements for the boys reached 0.747 for BMI, 0.748 for WC, and 0.779 for WHtR (*p *< 0.001) and 0.930 for BMI, 0.823 for WC, and 0.777 for WHtR (*p *< 0.001) for the girls.(Table 2).

### 3.3. Prevalence Rates of Overweight and Obesity

The prevalence of overfat estimated from %BF totaled 34.5% (95% confidence interval (CI) = 31.6%–37.4%) in the girls and 24.4% (95% CI = 21.9%–26.9%) in the boys. The boys exhibited a lower prevalence rate of overfat condition than the girls (*X*^2^ = 26.40,* p *< 0.001).

Among the boys, IOTF BMI-based reference for obesity produced lower prevalence rate of obesity than the other BMI-based references. Among girls, IOTF BMI-based reference for obesity had lower prevalence rate of obesity than WGOC BMI-based reference.

Among the girls, the overweight and obesity rates evaluated by WC references were similar with those evaluated by BMI-based references, whereas WHtR reference produced higher prevalence rate of overweight and obesity than BMI-based and WC references. Among the boys, the overweight and obesity rate evaluated by WC reference was lower than those evaluated by BMI-based references among boys, whereas WHtR reference produced overweight and obesity prevalence rate that was comparable to those determined by WHO and WGOC BMI-based references. The rates of overweight and obesity determined by WHtR reference were higher than those determined by IOTF and CDC BMI-based references (Table 3).

### 3.4. Sensitivity and Specificity of the References in Detecting Overfat Status

#### 3.4.1. SE and SP of the References for Obesity

As shown in Table 4, SE values of BMI-based references for obesity varied from 0.325 to 0.502 (WHO BMI: 0.502; CDD BMI: 0.448; IOTF BMI: 0.325; and WGOC BMI: 0.473) among the boys. All BMI-based references for obesity showed a very low SE in detecting overfat individuals among the girls. The values varied from 0.128 to 0.278. These findings indicate that about 68%–87% of overfat girls may be mislabeled as nonobese according to these BMI-based references for obesity. Chi-square tests showed that SE values for each reference for obesity for the boys were higher than those for the girls.

All BMI-based references for obesity used in the present study showed significantly high SP in detecting nonoverfat individuals in the study population. SP value for the girls reached 0.998 for four BMI-based references for childhood obesity. For the boys, the values varied from 0.971 to 0.993. YI indicated that the BMI-based references performed better in identifying overfat boys than girls. PPV (0.848–0.990) and LR+ (17.201–181.900) indicated that all BMI-based references for obesity performed well in assessing overfat individuals in both gender groups.

#### 3.4.2. SE and SP of the References for Overweight and Obesity

Table 4 presents the diagnostic performances of WC-based and BMI-based references for overweight and obesity in assessing overfat individuals. SE values of these references varied from 0.697 (WC reference) to 0.881 (WHtR reference) in boys and from 0.493 (CDC BMI reference) to 0.670 (WHtR reference) in girls. The findings indicate that 30.3% to 11.9% of overfat boys and 50.7% to 33.0% of overfat girls may be mislabeled as nonobese according to these references for overweight and obesity. Chi-square test showed that SE values of all the references for overweight and obesity were higher in boys than in girls.

All WC-based and BMI-based references for overweight and obesity showed high SP in both gender groups. The rates varied from 0.816 (WHtR reference) to 0.934 (WC reference) in boys and from 0.864 (WHtR) to 0.966 (CDC BMI reference) in girls. Similar YIs were observed in BMI-based and WC-based references for overweight and obesity in boys (0.630–0.697) and girls (0.448–0.533). PPV varied from 0.607 to 0.772 in boys and from 0.722 to 0.870 in girls. LR+ values reached 4.784–10.487 in boys and 4.920–14.648 in girls.

## 4. Discussion

A valid criterion for childhood obesity should identify overfat individuals correctly and those with high risk of morbidity [29]. In the current study, we investigated the diagnostic accuracy of four BMI-based and two WC-based references for childhood overweight and obesity in evaluating overfat individuals among Chinese children and adolescents. Results revealed that (1) prevalence rates determined by different BMI-based or WC-based references for childhood overweight and obesity varied; (2) the four BMI-based references for obesity exhibited low SE and high SP; (3) BMI- and WC-based references for overweight and obesity exhibited improved overall diagnostic performance in assessing overfat participants.

Body composition implies ethnic differences [30, 31]. Asian children and adolescents feature higher %BF than their European and African-American counterparts at the same BMI level [30, 31]. Therefore, the diagnostic accuracy of a universal classification system for childhood obesity may not correspond to a comparable level of body fat in all ethnic populations. International BMI-based references may overestimate or underestimate the prevalence of obesity in comparison with national definitions [32]. This situation is undesirable because it influences the evaluation of health services for those who most need intervention. In the current population, IOTF BMI-based reference for obesity produced a lower rate for childhood obesity than national BMI-based references. Similar findings were observed by a previous study [33]. The discrimination may be attributed to the different smoothing methods, reference populations, and use of BMI cut-off methods in developing different references for obesity. Several studies have shown that national BMI-based references for childhood obesity exhibit higher SE and similar SP in screening childhood and adolescent obesity compared with IOTF BMI approach [13, 34–36]. In a within-nation surveillance of childhood obesity prevalence, the lower SE of the IOTF BMI approach served as a limiting factor because it may lead to underestimation of the prevalence of overfat individuals in a national population. The IOTF BMI approach bears significance in comparing the prevalence of obesity across countries.

In the current study, BMI, WC, and WHtR exhibited strong positive correlations with %BF in boys and girls. Similar findings were presented by previous studies [37, 38]. The findings indicate the linear relationship between these anthropometric indices and %BF. However, the relationship cannot evaluate diagnostic performance of WC-based and BMI-based references for childhood overweight and obesity in assessing overfat.

In the current study, diagnostic performances of four BMI-based references for obesity were analyzed. The low SE and high specificity SP of these references in detecting overfat indicate that these references failed to achieve the optimal rates of SE for both genders. These results were consistent with the findings of previous studies [25, 33, 34, 37, 38]. However, the SE and SP of BMI-based references for childhood overweight and obesity in detecting overfat varied in these studies and showed large discrepancies due to the reference population, methods used in assessing %BF, adopted cut-offs from various references, and cut-offs for overfat status [33, 37].

Now, BMI-based references for overweight and obesity have been proved to show higher SE in evaluating participants with overfat than BMI-based reference for obesity in boys and girls [25, 36, 39]. We observed comparable SE and SP rates in screening overfat individuals according to the four BMI-based references for overweight and obesity. Thus, a BMI-based reference for childhood obesity may prove inefficient in estimating overfat individuals among Chinese children and adolescents.

Limited research have reported highly specific but less sensitive screening of overfat individuals by using WC-based references [37, 40]. These studies used WC-based references with different cut-offs for screening overfat individuals. Therefore, difficulty arises in comparison with findings between these studies and the current work. Reilly et al. observed no difference in the SE and SP between BMI- and WC-based indices in predicting cardiometabolic risks [13]. Overall, the WC-based references may be used to assess the prevalence of childhood obesity, which showed a comparable diagnostic performance in identifying excess fat to that of BMI-based references for overweight status and obesity in boys and girls.

The diagnostic performance of BMI-based references for overweight in screening overfat individuals can be improved by using BMI-based references for obesity. WC and WHtR references for overweight and obesity developed from local reference data showed similar diagnostic performances compared with BMI-based references for overweight. However, no references for overweight status and obesity performed well in identifying overfat individuals among the girls. Determination of an “ideal” cut-off value is almost always a trade-off between SE and SP. Ideally, a diagnostic test should possess both high SE and SP [41]. However, these characteristics are rarely achieved. For childhood obesity screening, researchers may desire to set a high SE by adjusting the cut-offs to reduce false-negative rates [33, 34].These results suggest that the optimal cut-offs of different anthropometric methods should be adopted to improve diagnostic accuracy in identifying overfat individuals among Chinese children and adolescents.

This study featured several limitations. Considering body fat distribution changes with puberty, the age range (9-19) is wide for measure WC. Although the study used health-related %BF cut-offs for defining overfat individuals, the cut-offs have received criticism on their sample size, reference population, selected health outcomes, and risk measures [23, 26].

More importantly, it is significant to explore the ability of WC, BMI, %BF, and WHtR to predict cardiometabolic status among individuals and establish risk-weighted cut-offs [27]. Among prepubertal children, previous study had developed percentage of fat and waist circumference cut-offs with the intention of defining obesity associated with cardiovascular disease risk [42], and WHtR can specify cardiometabolic risk on the basis of BMI percentile in US children and adolescents [11]. In Chinese adults, researchers used ROC curves to find optimal metabolic syndrome-weighted cut-offs of WC, BMI and WHtR; they found BMI and WC are more useful than WHtR for predicting nonadipose components of metabolic syndrome [43]. To evaluate these BMI, WC, and WHtR cut-offs, other health-related criteria, such as cardiovascular risk factors, among Chinese children and adolescents may be considered in the future. Further studies using a national representative sample are necessary to confirm the findings of the current study.

## 5. Conclusion

In conclusion, different references for childhood overweight and obesity produced different prevalence rates of childhood overweight and obesity in the current population. The diagnostic accuracy of BMI-based references for obesity was poorer than expected in Chinese children and adolescents. When BMI- and WC-based references for overweight and obesity were used to assess overfat status, these anthropometric index-based references showed improved diagnostic performance. The results suggest that further studies may focus on defining risk-weighted cut-off values for childhood obesity specific for a local population and ethnicity by using health-related overfat data.

## Figures and Tables

**Table 1:** Clinical characteristics of participants.

	Boys(n=1135)			Girls(n=999)		
	Median (range)	5^th^ percentile	95^th^ percentile	Median (range)	5^th^ percentile	95^th^ percentile
Age-z	0.07	-1.78	1.64	0	-1.64 *∗*	2.01 *∗*
	(-1.97-2.21)	(-1.97--1.68)	(1.44-2.21)	(-1.83-2.18)	(-1.83--1.59)	(1.59-2.18)
BW-z	-0.01	-1.72	2.23	-0.04	-1.76	2.15
	(-2.27-3.66)	(-2.27--1.54)	(1.75-3.66)	(-2.24-5.69)	(-2.24--1.52)	(1.68-5.69)
BH-z	0.28	-2.03	1.48	0.23	-2.37 *∗*	1.53 *∗*
	(-2.82-2.16)	(-2.82--1.83)	(1.34-2.16)	(-3.44-2.13)	(-3.44--2.07)	(1.33-2.13)
WC-z	-0.16	-1.43	2.59	-0.2	-1.51	2.43
	(-2.2-4.76)	(-2.2--1.25)	(1.98-4.76)	(-2.13-6.13)	(-2.13--1.33)	(1.88-6.13)
WHtR-z	-0.23	-1.32	2.44	-0.17	-1.42	2.51
	(-1.95-4.95)	(-1.95--1.17)	(1.97-4.95)	(-1.96-5.54)	(-1.96--1.24)	(1.97-5.54)
BMI-z	-0.18	-1.45	2.43	-0.17	-1.45	2.38
	(-2.09-4.37)	(-2.09--1.3)	(1.94-4.37)	(-2.04-5.71)	(-2.04--1.3)	(1.84-5.71)
%BF-z	-0.21	-1.39	2.42	-0.14	-1.57 *∗*	2.38
	(-1.95-4.14)	(-1.95--1.26)	(1.93-4.14)	(-2.25-5.77)	(-2.25--1.37)	(1.84-5.77)

Note: BW, body weight; BH, body height; WC, waist circumference; WHtR, waist-for-height ratio; BMI, body mass index; %BF, body fat percentage measured by BIA; *∗* p<0.05, boys versus girls.

**Table 2:** Age-adjusted correlation among the different indices of obesity.

	Boys (n=1135)			Girls (n=999)		
	BMI	WC	WHtR	BMI	WC	WHtR
%BF	0.747	0.748	0.779	0.930	0.823	0.777
BMI		0.918	0.877		0.930	0.813
WC			0.941			0.932

Note: BMI, body mass index; %BF, body fat percentage; WC, waist circumference; WHtR, waist-for-height ratio.

**Table 3:** Prevalence rates of obesity and overweight estimated by existing definitions for childhood overweight and obesity.

	%BF	BMI based definitions								WC based definitions	
		IOTF-BMI		CDC-BMI		WHO-BMI		WGOC-BMI		WHtR	WC
	Overfat	OB	OB & OW	OB	OB & OW	OB	OB & OW	OB	OB & OW	OB & OW	OB & OW
Boys (n=1135)	24.4% (21.9-26.9)	8.5% (6.9-10.1)	28.0% (25.4-30.6)	12.4% (10.5-14.3)	27.4% (24.8-30.0)	14.5% (12.5-16.6)	33.4% (30.7-36.1)	13.3% (11.3-15.3)	30.5% (27.8-33.2)	35.4% (32.6-38.2)	22.0% (19.6-24.4)

Girls (n=999)	34.5% (31.6-37.4)	4.4% (3.1-5.7)	20.0% (17.5-22.5)	6.6% (5.1-8.1)	19.2% (16.8-21.6)	6.9% (5.3-8.5)	22.3% (19.7-24.5)	9.7% (7.9-11.5)	21.8% (19.2-24.4)	32.0% (29.1-34.9)	24.0% (21.4-26.7)

Note: all data are presented as mean (95% confidential interval, 95%CI).

OB & OW, overweight and obesity; BMI, body mass index; %BF, body fat percentage measuring by BIA; WC, waist circumference; WHtR, waist-for-height ratio.

IOTF-BMI, International Obesity Task Force BMI reference; CDC-BMI, US Centers for Disease Control and Prevention BMI reference; WHO-BMI, World Health Organization reference; WGOC-BMI, Working Group on Obesity in China BMI reference.

**Table 4:** Diagnostic indices of different definitions for childhood obesity and overweight in screening excess %BF.

	References	SE		SP		YI		PPV		LR+	
		Boys	Girls	Boys	Girls	Boys	Girls	Boys	Girls	Boys	Girls
OB	CDC-BMI	0.448 (124/277)	0.191 (66/345)	0.980 (840/858)	0.998 (653/654)	0.428	0.190	0.978	0.985	22.567	125.113
	IOTF-BMI	0.325 (90/277)	0.128 (44/345)	0.993 (851/858)	0.998 (653/654)	0.318	0.126	0.938	0.978	46.408	83.409
	WGOC-BMI	0.473 (131/277)	0.278 (96/345)	0.977 (837/858)	0.998 (653/654)	0.450	0.277	0.868	0.990	20.265	181.900
	WHO-BMI	0.502 (139/277)	0.200 (69/345)	0.971 (832/858)	0.998 (653/654)	0.473	0.199	0.848	0.986	17.201	130.800

OW & OB	CDC-BMI	0.762 (211/277)	0.493 (170/345)	0.883 (757/858)	0.966 (632/654)	0.645	0.459	0.670	0.885	6.528	14.648
	IOTF-BMI	0.765 (212/277)	0.504 (174/345)	0.878 (752/858)	0.961 (628/654)	0.643	0.464	0.669	0.870	6.247	12.686
	WGOC-BMI	0.801 (222/277)	0.548 (189/345)	0.855 (733/858)	0.956 (625/654)	0.657	0.503	0.642	0.867	5.539	12.354
	WHO-BMI	0.845 (234/277)	0.542 (187/345)	0.856 (734/858)	0.945 (618/654)	0.678	0.487	0.619	0.839	5.027	9.847
	WC	0.697 (193/277)	0.533 (184/345)	0.934 (801/858)	0.914 (598/654)	0.630	0.448	0.772	0.757	10.487	6.229
	WHtR	0.881 (244/277)	0.670 (231/345)	0.816 (700/858)	0.864 (565/654)	0.697	0.533	0.607	0.722	4.784	4.920

Note: IOTF-BMI, International Obesity Task Force BMI reference; CDC-BMI, US Centers for Disease Control and Prevention BMI reference; WHO-BMI, World Health Organization reference; WGOC-BMI, Working Group on Obesity in China BMI reference; WC, waist circumference; WHtR, waist-for-height ratio.

SE, sensitivity; SP, specificity; YI, Youden's index; PPV, positive predictive values; LR+, likelihood ratio for positive test results.

## Data Availability

The anthropometric data used to support the findings of this study are available from the corresponding author upon request.
